# Supplementary material for: The Role of Plasma Extracellular Vesicles in Remote Ischemic Conditioning and Exercise-Induced Ischemic Tolerance
Source: Int J Mol Sci. 2022 Mar 19;23(6):3334. doi: 10.3390/ijms23063334 (PMC8951333; doi:10.3390/ijms23063334)
Supplement: Supplementary file 1 [file ijms-23-03334-s001.zip › ijms-1608423-supplementary.pdf]

Supplementary tables and figures

Table S1. Anthropometrics of human volunteers

Intervention	Age	Height	Weight	BMI
BFRRE	22	189	70.7	19.79
BFRRE	22	180	66.5	20.52
BFRRE	23	169	71	24.86
BFRRE	22	179	73.6	22.97
BFRRE	23	184	76	22.45
BFRRE	28	182	93.4	28.20
HLRE	22	176	75.5	24.37
HLRE	22	178	108.1	34.12
HLRE	23	179	62.6	19.54
HLRE	27	171	81.1	27.74
HLRE	27	182	83.7	25.27
HLRE	26	187	85.2	24.36
RIC	24	179	106.5	33.24
RIC	24	168	54.8	19.42
RIC	27	175	98.1	32.03
RIC	22	175	90	29.39
RIC	21	184	81.3	24.01
RIC	23	187	94.5	27.02
NIC	22	177	66.7	21.29
NIC	21	194	98.4	26.15
NIC	35	177	78.4	25.02
NIC	22	179	79.6	24.84
NIC	26	171	91	31.12
NIC	24	186	67.8	19.60

No significant differences between intervention groups

Table S2. EV Array antibodies and suppliers

Antibody	Supplier	Cat #	Clone
Annexin V	R & D Systems	AF399	
CD19	R & D Systems	MAB4867	4G7-2E3
CD146	Abcam	ab24577	P1H12
CD9	Ancell	156-020	
CD4	R & D Systems	MAB379	34930
CD63	Biorad	MCA2142	
EGFR	Ab Online	ABIN191750	
CD62 E	Thermo Scientific	MA1-22165	
LAMP2	R & D Systems	MAB6228	H4A3
CD81	Ancell	302-020	
TNF RII	R & D Systems	DY726	
Hsp90	Abcam	ab13495	IGF1
TNF RI	R & D Systems	DY225	
HLA ABC	Biolegend	311402	W6/32
CD142	R & D Systems	MAB2339	323514
CD13	R & D Systems	MAB3815	498001
CD3	BD Biosciences	555337	Hit3a
p53	Abcam	ab26-100	pAb240
Flotillin-1	Abcam	ab41927	
CD42a	LS Bio	LS-C45240	
Alix	Biolegend	634501	3A9
CD106	R & D Systems	MAB809	HAE-2Z
CTLA4	LS Bio	LC-C134750	ANC152.2/8H5
LFA1 (CD11a)	Ab biotec	250944	HI111
ICAM-1 (CD54)	eBioscience	BMS1011	R6.5
LRP-1	Abcam	ab20384	
HLA DR/DP/DQ	Svend Birkelund		HB-145
CD14	BD Biosciences	555396	M5E2
CD16	BD Biosciences	555404	3G8
CD56	BD Biosciences	559043	3G8
VEGFR2	Biolegend	359902	7D4-6
Lactadhedrin	Haematologic Technologies Inc.	BLAC-1200	
tPA	R & D Systems	AF7449	
Thrombospondin-1	R & D Systems	AF3074	
CD31 (PECAM-1)	R & D Systems	AF806	
PD-L1	Sino Biological	10084-R001	
N-Cadherin	Abcam	ab19348	8C11
TGFβ1	BD Pharmingen	555052	
CD235a (glycophorin A)	R & D Systems	MAB1228	
LAMP-1	R & D Systems	MAB4800	
CD25	BD Parmingen	555430	M-A251
OPRL1	Novus	NBP2-21065	poly
Osteopontin	R & D Systems	MAB14332	223112
Hsp70	R & D Systems	MAB1663	242707

Table S3. EV concentrations after purification and concentration

			CON			
Sample	14	5	7	16	21	46
Pre	1,58E+11	2,60E+11	4,43E+11	1,65E+11	1,74E+11	1,44E+11
5 min	2,15E+11	2,59E+11	3,56E+11	1,68E+11	1,40E+11	1,06E+11
30 min	9,25E+10	3,79E+11	2,58E+11	1,98E+11	1,40E+11	1,56E+11
6 weeks	4,29E+11	1,93E+11	3,27E+11	8,85E+10	1,17E+11	9,95E+10
			**RIC**			
Sample	15	30	6	8	23	32
Pre	1,26E+12	4,43E+11	3,90E+11	2,22E+11	3,84E+11	2,78E+11
5 min	1,03E+12	5,30E+11	7,70E+11	1,94E+11	3,91E+11	3,53E+11
30 min	1,43E+12	3,54E+11	5,70E+11	2,78E+11	3,68E+11	4,39E+11
6 weeks	1,22E+12	4,10E+11	8,80E+11	2,19E+11	2,08E+11	2,71E+11
			**BFRRE**			
Sample	2	4	33	36	39	42
Pre	2,35E+11	2,55E+11	1,83E+11	6,15E+11	2,50E+11	1,00E+11
5 min	3,24E+11	1,33E+11	1,08E+11	4,43E+11	2,18E+11	8,25E+10
30 min	2,28E+11	2,00E+11	1,39E+11	4,62E+11	1,87E+11	1,13E+11
6 weeks	1,00E+11	1,32E+11	1,12E+11	3,38E+11	2,14E+11	1,12E+11
			**HLRE**			
Sample	26	40	10	1	38	3
Pre	3,67E+11	1,69E+11	3,45E+11	2,81E+11	2,02E+11	8,80E+10
5 min	3,96E+11	2,00E+11	3,07E+11	3,10E+11	3,29E+11	1,15E+11
30 min	2,10E+11	3,76E+11	3,89E+11	2,77E+11	2,31E+11	6,80E+10
6 weeks	2,23E+11	2,86E+11	4,31E+11	2,62E+11	1,53E+11	7,70E+10

The EV concentration was adjusted to the EV preparation with the lowest concentration for each subject.

Table S4. EV Array results of the 8 most pronounced surface markers.

NIC				
Marker	*5 min vs Pre*	*30 min vs Pre*	*6 weeks vs Pre*	*P-value*
CD3	-0.09	0.10	-8.88E-16	0.27
CD9	0.03	-0.07	0.03	0.77
CD16	-0.15	-0.15	-0.15	0.33
CD25	-0.07	0.03	-0.03	0.51
CD31	-0.02	0.00	-8.88E-16	1.00
CD81	0.00	0.00	0.00	1.00
ICAM-1	0.18	0.15	0.15	0.39
LRP-1	0.10	-0.07	0.00	0.18
**RIC**				
Marker	*5 min vs Pre*	*30 min vs Pre*	*6 weeks vs Pre*	*P-value*
CD3	0.06	0.11	0.11	0.49
CD9	0.05	-0.14	0	0.18
CD16	0.03	0.01	-0.02	0.95
CD25	0	0	0	1
CD31	-0.05	-0.07	0	0.78
CD81	-0.07	-0.04	-0.09	0.83
ICAM-1	0.04	0.12	-0.04	0.39
LRP-1	-0.05	0.02	0.04	0.53
**BFRRE**				
Marker	*5 min vs Pre*	*30 min vs Pre*	*6 weeks vs Pre*	*P-value*
CD3	0	0.11	-0.02	0.33
CD9	-0.11	-0.19	-2.22E-15	0.1
CD16	0.01	-0.1	-0.04	0.47
CD25	0.08	0.04	0.12	0.13
CD31	0	0.02	0.08	0.81
CD81	0.18	0.25	0.05	0.05*
ICAM-1	-0.12	-0.12	-0.25	0.07
LRP-1	-0.04	-0.02	0.07	0.33
**HLRE**				
Marker	*5 min vs Pre*	*30 min vs Pre*	*6 weeks vs Pre*	*P-value*
CD3	-0.07	0.05	0.02	0.49
CD9	-0.05	-0.02	4.44E-16	0.95
CD16	-0.06	-0.1	0.05	0.23
CD25	0.13	0.09	0.13	0.06
CD31	0.01	-0.04	-0.18	0.13
CD81	0.07	0.13	0.18	0.29
ICAM-1	-0.07	-0.14	-0.14	0.37
LRP-1	0.04	0.04	-0.06	0.36

The table summarized the pre- and post-intervention EV surface markers from the EV Array data (comparison of Log2-fold changed data). * Asterisk indicate statistically significance between groups, p=0.05.

Figure S1. Gating of flowcytometer

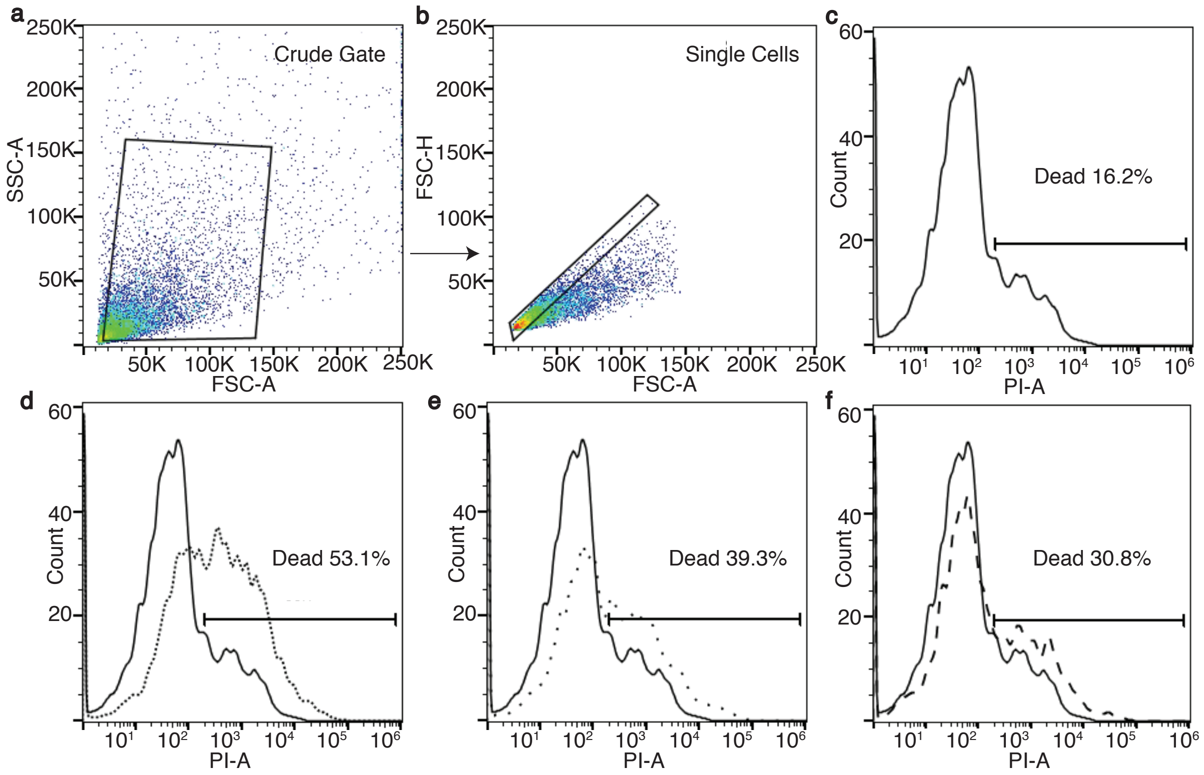

The cells were gated by their FSC-A x SSC-A scatter from which single cells were gated by their FSC-A x FSC-H scatter. Based on a negative sample (a normoxic sample without propidium iodide), a third gate was created to gate the dead cells from the single cells. The third gate was used to determine the percentage of dead cells in each sample.

Figure S2. Blood gas measurement for the acute in vivo study

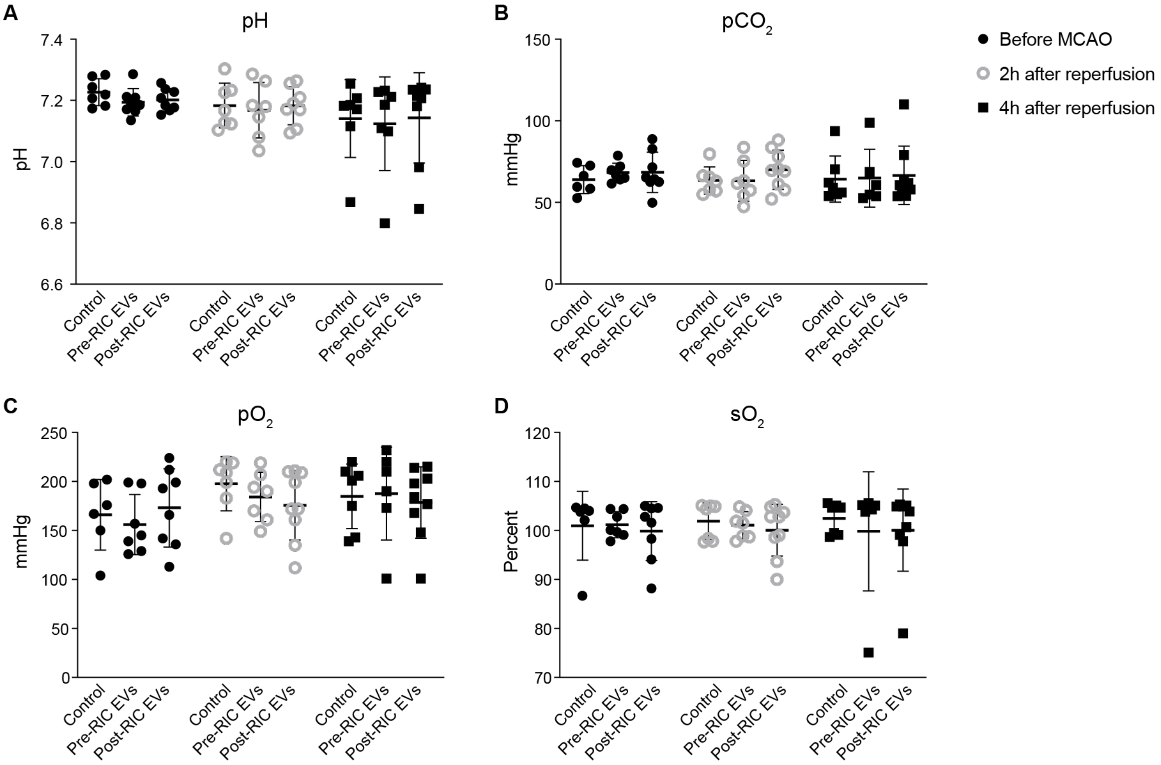

Blood gas measurement from the blood samples taken from the femoral artery before MCAO surgery, at 2 hours after reperfusion and 4 hours after reperfusion respectively. A) PH measurement. B) Partial pressure of CO2 C) Partial pressure of oxygen D) Saturation of oxygen. No significant group differences were found in these blood gas measurements. Data are expressed as mean with SD.

Figure S3. Angiogenic potential of post intervention EVs.

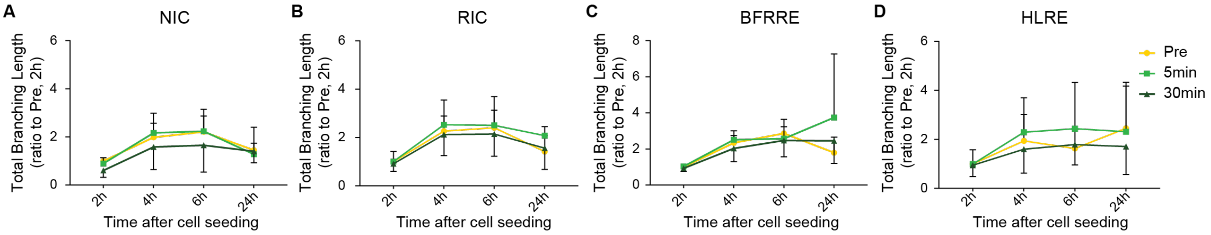

The branching length of HBMECs cultured with EVs from human volunteers subjected to NIC (A), RIC (B), BFRRE (C), or HLRE (D) at different timepoints post seeding. Each timepoint is normalized to the branching length at 2h post seeding in the pre-EV treated cultures. No significant differences in tube formation over time between pre-, 5-, or 30-min post intervention EVs or between interventions and the non-intervention control (NIC) EVs was found. The differences in branching length were largest, although not significant, in BFRRE pre- vs. 5 min at 24h (p-value = 0.10) and RIC pre- vs. 5 min post intervention at 24h (p-value = 0.24).

Figure S4. IgG extravasation at 4 h reperfusion in the tMCAO mice.

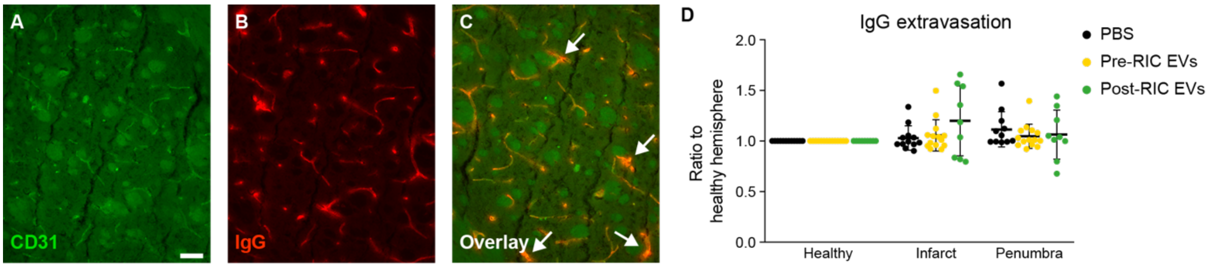

Representative stainings in the infarcted area of the striatum. Anti-CD31 stains endothelial cells lining the vessels (green, A), anti-mouse IgG (red, B), merged picture of CD31 and IgG staining (C). White arrows point to sites of blood brain barrier leakage. Comparing IgG extravasation in the infarct or penumbra area to the healthy hemisphere (D) showed significantly more extravasation in the penumbra compared to the healthy hemisphere (p=0.041). No group differences were found in IgG extravasation in any of the areas. Data are presented as mean±SD. Scalebar: 50 µm.
